# Xeno- and transgene-free reprogramming of mesenchymal stem cells toward the cells expressing neural markers using exosome treatments

**DOI:** 10.1371/journal.pone.0240469

**Published:** 2020-10-13

**Authors:** Luis Sebástian Alexis Valerio, Kiminobu Sugaya

**Affiliations:** 1 Burnett School of Biomedical Sciences, College of Medicine, University of Central Florida, Orlando, FL, United States of America; 2 Institute for Scientific Research and Technology Services (INDICASAT), Panama City, Republic of Panama; 3 Department of Biotechnology, Acharya Nagarjuna University, Guntur, India; Lewis Katz School of Medicine at Temple University, UNITED STATES

## Abstract

Neural stem cells (NSCs), capable of self-renew and differentiate into neural cells, hold promise for use in studies and treatments for neurological diseases. However, current approaches to obtain NSCs from a live brain are risky and invasive, since NSCs reside in the subventricular zone and the in the hippocampus dentate gyrus. Alternatively, mesenchymal stem cells (MSCs) could be a more available cell source due to their abundance in tissues and easier to access. However, MSCs are committed to producing mesenchymal tissue and are not capable of spontaneously differentiating into neural cells. Thus, the process of reprogramming of MSCs into neural cells to use in clinical and scientific settings has significantly impacted the advancement of regenerative medicine. Previously, our laboratory reported trans-differentiation of MSCs to neural cells through the induced pluripotent stem (iPS) cells state, which was produced by overexpression of the embryonic stem cell gene NANOG. In the current study, we demonstrate that treatment with exosomes derived from NSCs makes MSCs capable of expressing neural cell markers bypassing the generation of iPS cells. An epigenetic modifier, decitabine (5-aza-2'-deoxycytidine), enhanced the process. This novel Xeno and transgene-free trans-differentiation technology eliminates the issues associated with iPS cells, such as tumorigenesis. Thus, it may accelerate the development of neurodegenerative therapies and *in vitro* neurological disorder models for personalized medicine.

## Introduction

Neural stem cells (NSCs) residing in the subventricular zone and granule layer of the dentate gyrus of the hippocampus. They are the ideal cell source for the neuro-regeneration therapies, considering they are capable of self-renewal and spontaneous differentiation into neural cells, neurons, astrocytes, and oligodendrocytes. However, a highly risky and invasive procedure is required to obtain NSCs from a donor because they localize within the deep brain. Cellular reprogramming may overcome this issue by providing an alternative way to produce the neural cells from the somatic cells.

Somatic stem cells, such as mesenchymal stem cells (MSCs) are promising materials for the reprogramming since they possess multipotency and self-renewal ability, and are abundant in many tissues, such as bone marrow, adipose tissue, and blood. We have reported that epigenetic modifications [1], or overexpression of embryonic stem (ES) cell gene [2–3] induced trans-differentiation of MSCs to neural cells. We showed NANOG induced expression of other embryonic transcription factors, such as Oct3/4 and Sox2, to increase the potency of the cells [2–3]. A similar outcome was later achieved by Yamanaka's group who created induced pluripotent stem (iPS) cells from fibroblasts through the overexpression of OCT3/4, SOX2, and other tumor genes [4]. These iPS cells are an unlimited source of autologous cells that can produce any tissue without any ethical concerns or immunological rejection problems associated with ES cells. However, iPS cells tend to produce tumors because of the tumorigenic nature of the transgenes used. However, this technology is still worth using for modeling diseases and drug screenings in vitro. To overcome the issues, many researchers attempted to create iPS cells in a safer and faster manner through various methods [5–9].

Nevertheless, all these methods are still lengthy, unsafe, cumbersome, since the mechanism behind reprogramming is not yet well understood, limiting its improvement. Many researchers have been trying to directly convert somatic cells to induced neural stem-cell-like cells (iNSCs) with a process known as "direct reprogramming," bypassing the pluripotent state to avoid risks associated with iPS cells by a variety of ways [10–15]. The direct reprogramming strategy may be favorable for cell therapy and modeling of neurodegenerative diseases because it may directly produce NSCs from adult cells without issues associated with iPS cells. Previous studies have demonstrated that iNSCs were able to survive, migrate, proliferate, functionally integrate into the host brain, and improve behavior in neurodegenerative disorder models [16–18], indicating their potential for use as transplantation material for neuroregenerative cell therapies. Nevertheless, further efforts are required to establish xenogeneic free, safer, and more efficient technology for the production of iNSC.

Although MSCs are the leading choice for cell therapies due to their regenerative properties. However, their use is limited to the production of connective tissue since they do not differentiate into any cell type outside the mesoderm layer. A major obstacle to the reprogramming of somatic stem cells to commit to the cell types from a different germ layer is their epigenetic memory in the form of DNA methylation, limiting the potency of the cells. Previous studies showed that epigenetic modifications with 5-Bromo-2'deoxyuridine (BrdU) [1] or decitabine (5-aza-2'deoxycytidine) [19], increased the potency of MSCs. Decitabine induces DNA demethylation by blocking DNA methyltransferase and prevent differentiation of neural progenitors [20]. Therefore, decitabine treatment may erase the epigenetic memory and facilitate the trans-differentiation of MSCs.

We previously showed that epigenetic modification and overexpression of NANOG increased the potency of MSCs. The epigenetically enhanced MSCs exposed to neural cell environments, such as a co-culturing with neural cells or transplantation to the brain, trans-differentiate into neural cells [1–3]. The mechanism of this neural differentiation could involve the transmission of cell-lineage regulation factors from the neural cells to the modified MSCs by exosomes, vesicles known to play a role in cell-to-cell communication. Exosomes are the endosomal vesicles. They are released into the extracellular space upon fusion with the plasma membrane. These particles less than 100nm in diameter contain nucleic acids, proteins, and other substances specific to their cell of origin and the environment where they belong. Takeda et al. reported that exosomes from rat pheochromocytomas transdifferentiated MSCs to neuron-like cells [21]. The pheochromocytomas show neural cell characteristics after treatment with neuronal factors. This result indicates that exosomes derived from human NSCs may induce neural cell lineages to MSCs. However, exosomes from cancer cells may transfer tumorigenic factors to recipient cells [22]. Thus, we hypothesized that exposing MSCs to exosomes derived from human NSCs may induce trans-differentiation of MSCs to NSCs. We also hypothesized that decitabine treatment increases the efficacy of the trans-differentiation.

Here, we report the direct reprogramming of MSCs toward neural cell linage by epigenetic modifications. Our results indicate that exosomes transfer factor(s) to modify the cell linage of MSCs, and treatment with decitabine accelerates the process. This xeno-free and transgene-free technology to produce iNSCs from MSCs that may provide cell materials for in vitro personalized medicine models and autologous cell transplantation therapies for neurological diseases in an effective, fast, and safe manner.

## Methods

### Cell culture

Human adipose tissue-derived MSCs (Lonza) were expanded in Xeno-free MSC media containing DMEMF12 (Invitrogen), 10% KSR (Invitrogen), 5% Glutamax (Invitrogen), 5% NNEA (Invitrogen), 1% antibiotics and antimycotics (Invitrogen) in T75 tissue culture treated flask coated with 0.25ug/mL recombinant human laminin (rhLaminin) commercially available as iMatrix-511 (Nippi).

Human fetal-derived NSCs (Cambrex) were expanded in Xeno-free NSC media containing DMEMF12 (Invitrogen) supplemented with B27 (1:50 Invitrogen), basic Fibroblast Growth Factor (bFGF 10μg/mL, R&D Systems), Epidermal Growth Factor (EGF 20ug/mL, R&D Systems), Heparin (1000U/mL, Sigma) and 1% antibiotics and antimycotics (Invitrogen).

Human-induced pluripotent stem (iPS) cell line CW50064 (Cellular dynamics) were grown using mTeSR media (Stem Cell Technology) on 12 well plates coated with PL Matrix (PL bioscience).

### Isolation of exosomes

Ten mL of spent media from confluent cultures of NSC and iPS cells were collected in 50 mL falcon tubes. The spent media was centrifuged at 10000xg for 30 minutes to remove cell debris. Exosomes were isolated from conditioned culture media using a modified PEG-NaCl precipitation method. In brief, 10 ml of supernatant was used to precipitate exosomes by adding 5 ml of 20% PEG and 200 μl of 7.5 M NaCl and subsequent overnight incubation at 4˚C. The following day, the supernatant was centrifuged at 10000xg for 60 minutes and the exosome pellet was re-suspended in 1x PBS (pH 7.4, sans Calcium and Magnesium). Using CD63 conjugated magnetic beads [Invitrogen by Thermo Fisher Scientific Exosome—Human CD63 Isolation/Detection (from cell culture media), Ref- 10606D], the exosomes were further purified following the manufacturer’s protocol.

### Treatment of MSCs to produce iNSCs

**Fig 1:**
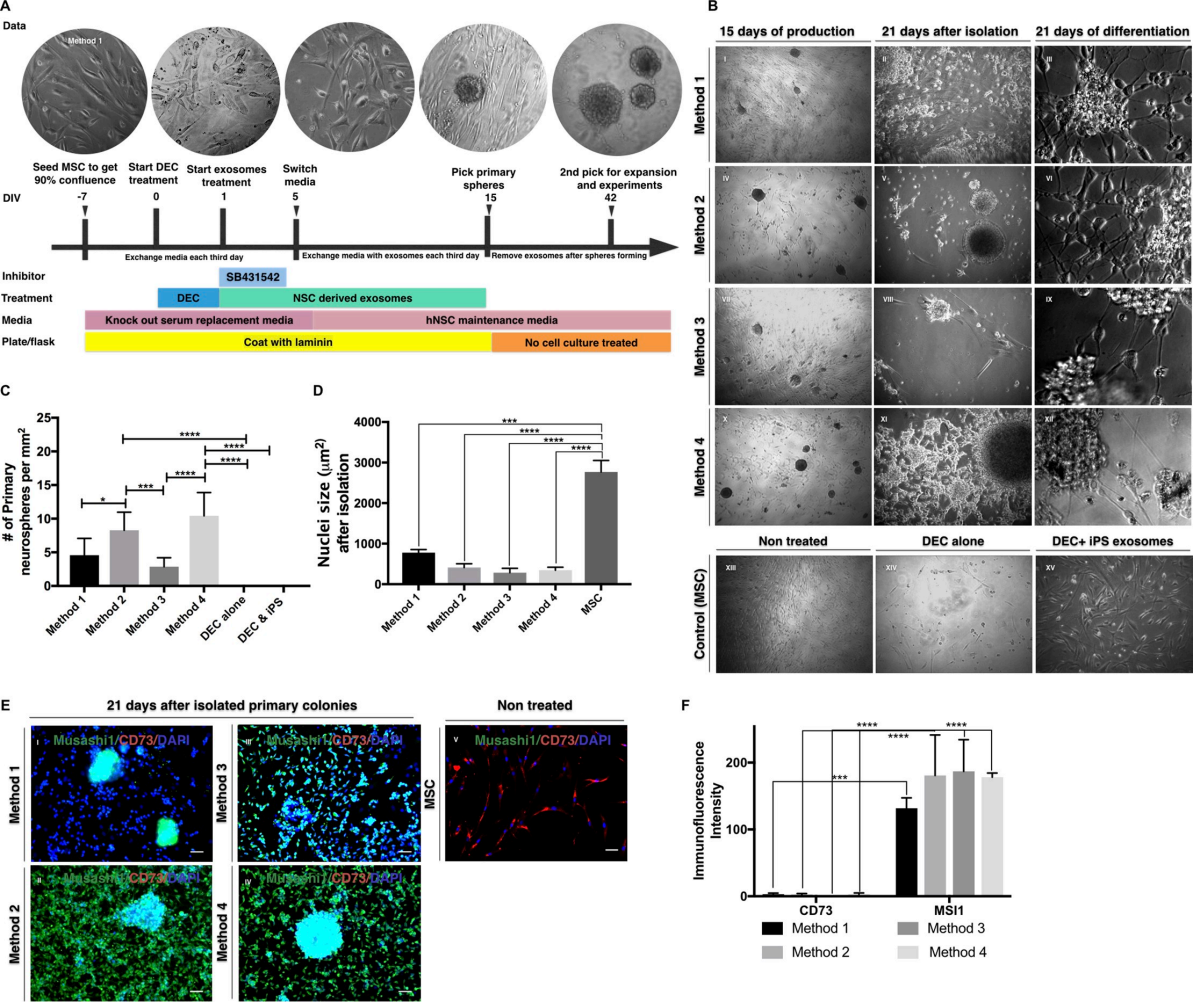
Generation of cells expressing neural markers under Xeno-free conditions. Panel A shows the schematic procedure to produce iNSCs from MSCs. Method 1; treatment with NSCs derived exosomes alone. Method 2; treatment with NSCs derived exosomes in the presence of 10μM decitabine. Method 3; treatment with exosomes derived from both NSCs and iPS cells. Method 4; treatment with exosomes derived from both NSCs and iPS cells in the presence of 10μM decitabine. Panel B shows phase-contrast microscopic images for 3 different stages, production (15 DIV), Isolation (21 DIV), and differentiation in various methods 1–4. XIII. No treatment: without any treatment. XIV. Treatment alone: decitabine treated MSCs. XV. Treatment + iPS exosome: MSCs treated with iPS cells derived exosome with decitabine treatment are shown as controls. Panel C shows the number of primary neurospheres formed at 21 DIV. The decitabine treated cells developed significantly higher numbers of spheroids as compared to those without the treatment. Without NSCs derived exosome treatment, any spheroid formation was not observed. Panel D shows the differences in the nucleic size of the cells at 21 DIV. The iNSC produced in all four methods showed significantly reduced nuclei size as compared to the naive MSCs. Panel E shows immunocytochemistry of iNSCs and MSCs (control) after differentiation for NSC marker, Musashi (MSI1, green), and MSC marker (CD73, Red). The nuclei were counterstained with DAPI (Blue). The scale bar is 50μm. Panel F shows semi-quantitative fluorescence intensity measurement by Image J. *P<0.05, **P<0.01, ***P<0.005, ****P<0.0001.

As shown in Fig 1A, MSCs were plated at a density of 25,000 cells per well on 12-well plates, previously coated with rhLaminin, and Xeno-free MSC media described previously. Experimental groups were treated with decitabine (10μM, Sigma-Aldrich) for 24 hours in Xeno free MSC media. Then, we washed the cells with Xeno free MSC media to remove decitabine. We treated MSCs with exosomes from iPS cells and/or NSCs and a TGF-*β* inhibitor (50nM, SB431542, Tocris Bioscience) for five days. Then media was replaced with exosomes derived from NSCs and/or iPS cells in NSC media every three days. At 15 days in vitro (DIV), exosomes were removed from the NSC media when neurospheres started to form in the culture. Neurospheres were transferred to a T75 nonadherent flask on 21 DIV for expansion in suspension culture using NSC media. For immunocytochemistry, we plated 4–6 neurospheres to 8-well chamber slides coated using rhLaminin for six hours.

### Differentiation of iNSCs derived from MSCs

iNSCs were plated at 5000 cells per well on several 8-well chamber slides or 48-well plates, previously coated using recombinant human laminin. Then the iNSC were incubated neural differentiation media containing Neurobasal Media (Invitrogen) supplemented with N2 (1X, Invitrogen), B27 (1:50, Invitrogen), and 1% antibiotics and antimycotics (Invitrogen) for 21 days.

### Immunocytochemistry

After fixation using 4% paraformaldehyde (PFA, Sigma) for 20 min at room temperature, we washed the cells three times using PBS (Sigma) with 1% BSA (Sigma). Then, cells were permeabilized using 0.3% Triton-X (Fisher Scientific) and 10% normal donkey serum (Jackson ImmunoResearch) for one hour. Then, samples were washed with PBS (Sigma) three times. The samples were incubated overnight at 4˚C with the primary antibodies against Pax6 (1:250 Invitrogen), GFAP (1:1000 Invitrogen), NeuN/Fox3 (1:1000 Invitrogen), βIII tubulin (1:1000 Invitrogen), Sox1 (1:1000 Invitrogen), Musashi1 (1:1000, Invitrogen), CD73 (1:1000 R&D systems), Nanog (1:1000 R&D systems), Sox2 (1:1000 R&D systems), Oct4 (1:1000 R&D systems), FABP4 (1:1000 R&D systems), aggrecan (1:1000 R&D systems), osteocalcin (1:1000 R&D systems), nestin (1:1000 Chemicon), DCX (1:1000 Chemicon), synaptophysin (1:1000 Chemicon), and CD90 (FITC conjugated, Biolegend). The next day, cells were washed with 0.1% BSA/PBS (Sigma) for three times at room temperature and incubated with secondary antibodies (1:500) conjugated with FITC or TRITC for two hours at room temperature. After the incubation, cells were washed three times using PBS and then mounted with a water-based mounting solution containing DAPI (Vector Laboratories). For the labeling of exosomes, we used the ExoGlow-protein EV labeling kit green (System Biosciences) according to manufacturer instructions. For imaging, we used the Zeiss 719 confocal microscope and the Zeiss AxioObserver microscope and objectives for 10X and 20X magnification. Then we used Image J to measure immunostaining intensities.

### Quantification of fluorescence intensities

We used Image J (NIH) to measure the fluorescence intensities of the immunostained samples. We randomly took images triplicated area from the triplicate fluorescence-immunostained samples. The fluorescence intensity was converted into an 8-bit digital scale. The mean of the 8-bit level of the randomly selected area was measured as the data. To detect autofluorescence and other nonspecific background signals, we took images from samples incubated without the primary antibodies. The background signal values were subtracted from the measured values of the fluorescence images to obtain the net fluorescence intensity. The semiquantitative image data were statistically analyzed by ANOVA and followed by post hoc Tukey test ran on GraphPad Prism 7 software.

### Flow cytometry analysis

The cells were mechanically dissociated and washed with PBS. After one wash with staining buffer (0.1% BSA/PBS, Sigma), 100000 cells were incubated with PE-conjugated antibodies against CD105 (5:100, Biolegend), CD90 (5:100, Biolegend), CD73 (5:100, Biolegend), CD45 (5:100, Biolegend) and Isotype control PE-mouse IgG1 (5:100, Biolegend). For the exosomes, we used the ExoGlow-protein EV labeling kit green (System Biosciences) according to manufacturer instructions. Then, we analyzed the freshly stained samples with the FACS CitoFlexS flow cytometer (Beckman). We detected autofluorescence background signals from the same samples without labeling and subtracted it from the data. The flow cytometry data were analyzed using FloJo (FloJo LLC).

#### Gene expression analysis

We isolated total RNA using the RNAeasy micro kit plus (Qiagen) according to the manufacturer's instruction. Then, we created cDNA using the Superscript III first-strand synthesis kit (Invitrogen). Real-time two-step RT-PCR was performed using a Fast SYBR green PCR mix (Applied Biosystems) and analyzed by the delta-delta Ct analysis method. We calculated relative expressions of undifferentiated and differentiated marker genes against GAPDH expression as the reference gene. The primer sets used were: MSI1 F:5'-GCCCAAGATGGTGACTCG-3' R:5'-ATGGCGTCGTCCACCTTC-3'; NESTIN F:5'-CGTTGGAACAGAGGTTGGAG-3 R:5'-TCT GGGGTCCTAGGGAATTG-3; PAX6 F:5'-TGGTATTCTCTCCCCCTCCT-3 R:5'-TAAGGATGTTGAACGGGCAG-3; βIII-tubulin F:5'-ATGAGGGAGATCGTGCACAT-3' R:5'-GCCCCTGAGCGGACACTGT-3; MAP2 F:5'-CAGGTGGCGGACGTGTGAAAATTGAGAGTG-3 R: 5'-CACGCTGGATCTGCCTGGGGACTGTG-3; SHH F:5'-AGAAACTCCGAGCGA TT-3 R:5'-CCTCGTAGTGCAGAGACTCC-3; FOXG1 F:5'-TGTTGACTCAGAACTCGCTGG-3 R:5'-CTGCTCTGCGAAGTCATTGAC-3; GAPDH F:5'-GTCATACCAGGAAATGAGCT-3' R:5'-TGACCACAGTCCATGCCATC-3' [23–26].

#### Statistical analysis

We used the one-way ANOVA and followed by a post hoc test, Newman-Keuls, to analyze the statistical defiance between the groups. We considered a p-value of less than 0.05 as statistically significant.

## Results

### Generation of iNSCs from human MSCs

Fig 1A shows the schematic procedure to produce iNSCs from MSCs. We compared the trans-differentiation of MSCs with the following four different exosome treatments under TGF-*β* inhibition: Method 1; NSCs derived exosomes treatment alone, Method 2; NSCs derived exosomes treatment in the presence of 10μM decitabine, Method 3; treatment with exosomes derived from both NSCs and iPS cells, Method 4; treatment with exosomes derived from both NSCs and iPS cells in the presence of 10μM decitabine. As shown in Fig 1B, a few MSCs changed their morphology to the neurosphere by NSCs exosomes and a TGF-*β* inhibitor treatment (Method 1) at 15 days in vitro (DIV). Although primary colonies could be ready to pick at this time point, we let them grow more and isolated at 21 DIV. The decitabine treatment significantly increased numbers of the primary sphere formations in Methods 2 (p = 0.0273) and 4 (p = 0.0004) as compare to Methods 1 and 3, respectively (Fig 1C). The Control group without any exosome treatment did not show spheroid formation. This result indicates that decitabine increased the transdifferentiation efficacy from MSC to iNSC. On the other hand, there is no significant difference between Method 1 and 3, meaning that iPS cells derived exosome treatment did not affect the transdifferentiation of MSCs.

We transferred the resulting neurospheres to the NSC media and cultured for another 21 days. As shown in Fig 1D, the cells forming typical neurosphere morphologies showed significantly smaller nuclei size (p<0.005) compared to that of the original MSCs. Also, the shape of the cell displayed a small rounded cell body that is similar to NSCs under the same culture condition. These cells expressed an NSC marker, Musashi1 (MSI1), but not an MSC marker, CD73 in Methods 2–4 (Fig 1E and 1F). Some of the cells resulted in Method 1 expressed both MSI1 and CD73, indicating a mixed population of transdifferentiated cells and MSCs. Thus, we further purify them by selecting the neurospheres again during the expansion. After another 21 days expansion, neurospheres in Method 1 did not show MSCs markers (CD105, CD90, and CD73) expression (S1 Fig), suggesting that the culture in NSC media may selectively expand iNSCs over MSCs. These results indicate that treatment with exosomes derived from NSCs under suppression of TGF-*β* signaling was able to convert MSCs to iNSCs, the cells having NSC morphology and markers. Decitabine treatment increased the conversion efficiency. Since we used iPS cells reprogrammed from human fibroblasts using ectopic expression of the Yamanaka factors, exosomes derived from the iPS cells may carry those factors. Still, it did not affect the conversion of MSCs to iNSCs.

### Expansion and characterization of the iNSCs derived from MSCs

**Fig 2:**
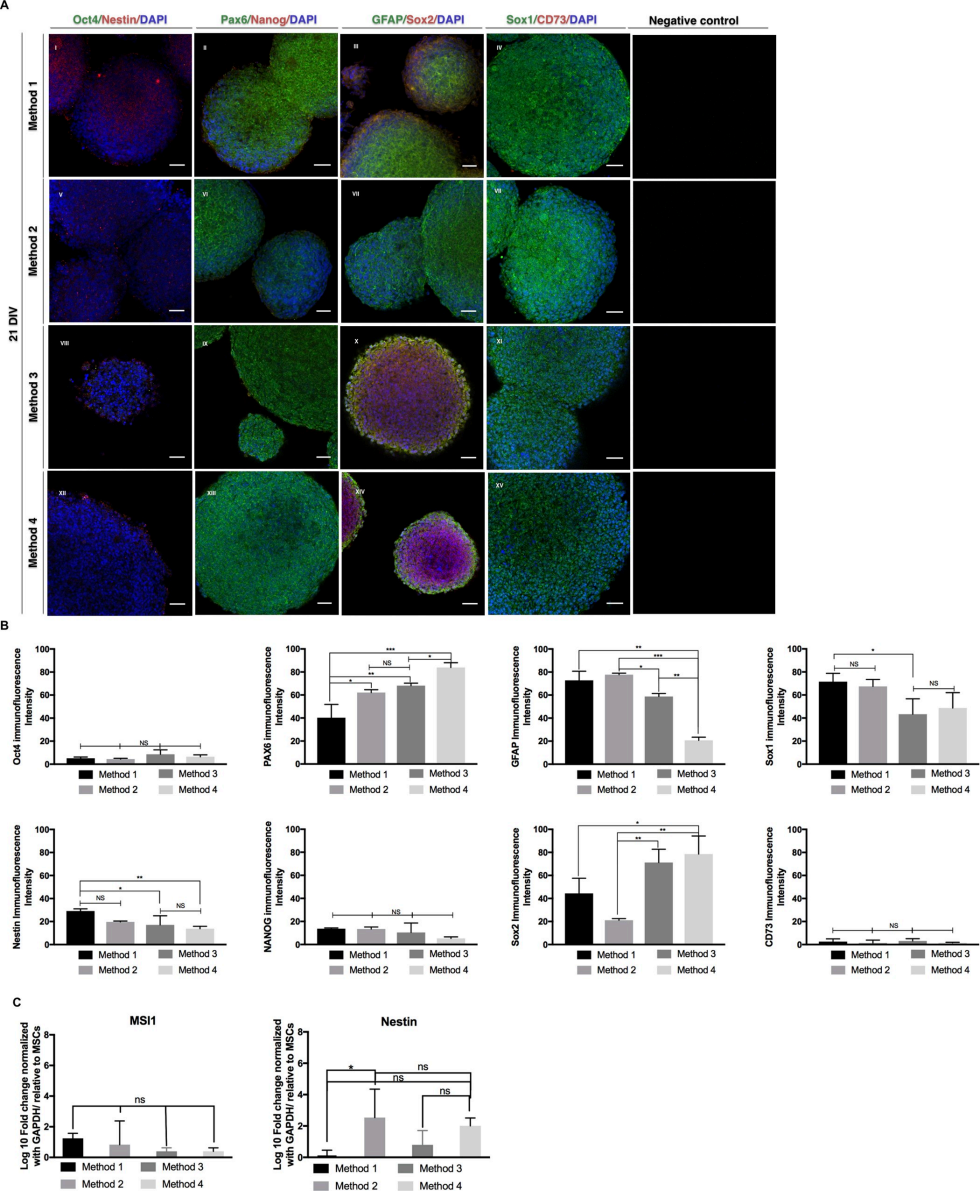
Characterization of the iNSCs. The iNSCs forming spheroids after 21 DIV expressed NSC markers, Nestin, Pax6, GFAP, after expansion in the suspended cell culture condition (panel A). While the iNSCs were negative for stem cell markers, Oct4 Nanog, and CD73 (panel A). Panel B shows a semiquantitative analysis of the fluorescence intensities. Panel C shows the gene expression Musashi1 (MSI1) and Nestin. All nuclei were counterstained with DAPI. The scale bar is 50μm. *P<0.05, **P<0.01, ***P<0.005.

We expanded iNSCs in suspension culture with NSC media with passage of mechanical dissociation of the spheroids to purify the iNSC population. We did not detect any fibroblast-like adherent cells after four passages. The iNSCs expressed NSCs markers, such as Sox1, Sox2, Nestin, Pax6, but not ES cell markers (Oct4 and Nanog) or MSC marker (CD73) in all for methods after the 21 days of expansion (Fig 2A and 2B). There was a significant increase in Sox2 immunoreactivity in iNSCs produced with Method 3 (p = 0.0036) and 4 (p = 0.0015) compared to the one created with Method 2. We detected significantly increased nestin protein expression levels in iNSCs produced with Methods3 (p = 0.0339) and 4 (p = 0.0091) compared to the one created with Method 1. These results indicate that the addition of iPS cells derived exosomes may increase NSC marker expression. However, Sox2 might have been a carryover from iPS cell exosomes since it is a pluripotency marker.

Additionally, we conducted a population analysis of the adherent cultures of the iNSCs. The S2 Fig shows the morphology for iNSCs expanded with adherent conditions on laminin-coated culture flasks after 21 days. The population of cells expressing NSC markers, MSI1 (p = 0.0176), Sox1 (p = 0.0107), and an ES cell marker Nanog (p = 0.0046) was increased in iNSCs produced with Method 3 as compared to the Method 1. There was also further increase in an NSC marker, Sox1 (p = 0.0003), and ES cell markers, Oct4 (p = 0.0017), and Nanog (p = 0.0152) in iNSCs produced with Method 4 as compared to the Method 3. These results suggest that adhesion cultures showed different characteristics, especially in the iNSCs treated with exosomes derived from iPS cells. Although the properties of iNSC expanded in adhesion conditions differ from those in suspension conditions, they were capable of self-renewal and expansion for multiple passages.

#### In vitro differentiation of iNSCs

**Fig 3:**
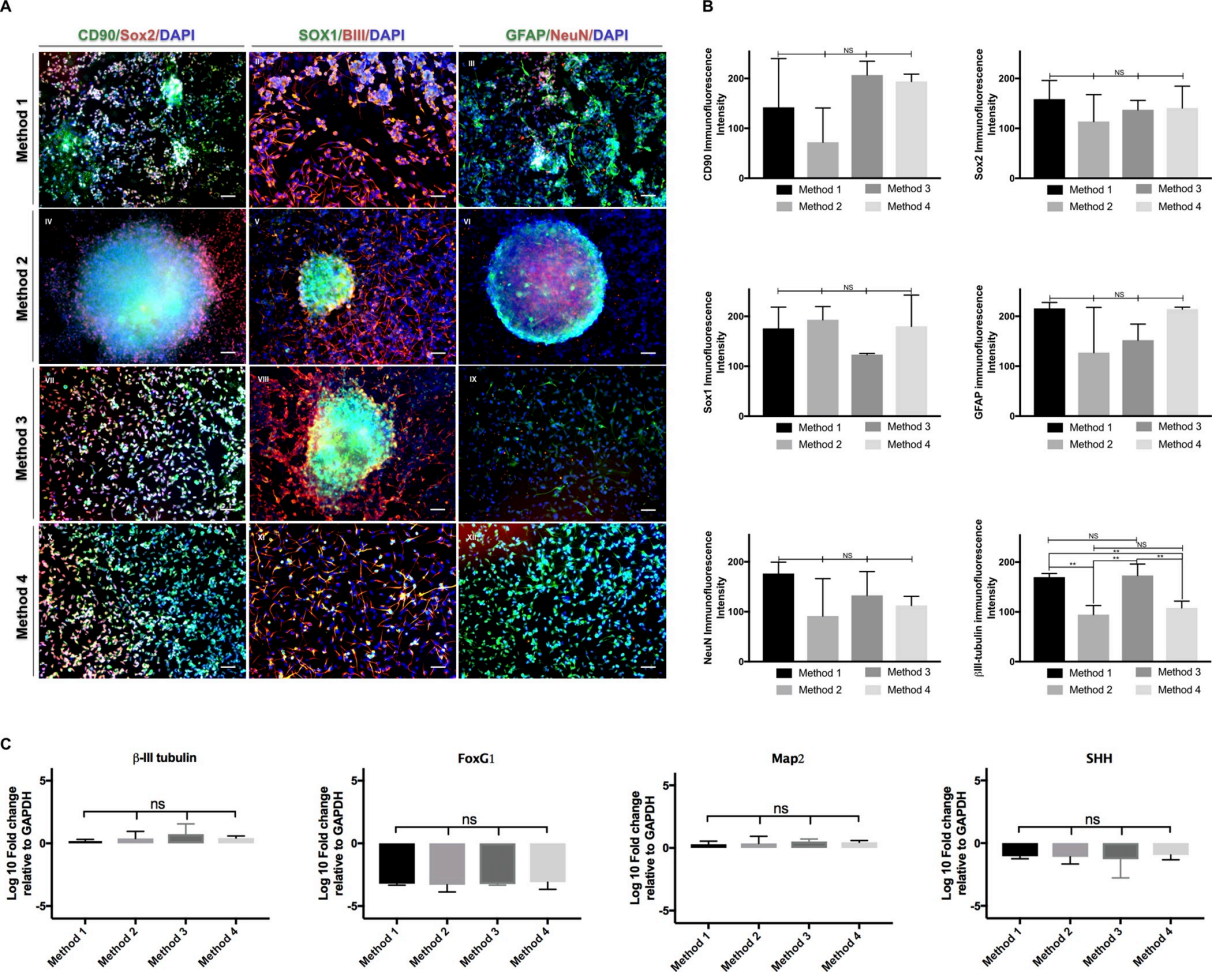
Differentiation of iNSCs under serum-free conditions. The resultant cells of the four methods expressed neural cell markers, βIII-tubulin, GFAP, and NeuN/Fox3a, which is more evident in the surrounded areas of the spheres, although its expression may be inside the sphere as well. (A) On the other hand, the NSC markers Sox2, Sox1, and CD90 were observed inside the spheres (A). Panel B shows the difference between the immunofluorescence intensities of the immunostaining of panel A. It is important to note that cultures that received decitabine (method 2 & 4) showed significantly less fluorescence intensity signals for βIII-tubulin, suggesting differentiation may be delayed and stemness retained for these cell cultures. (C) shows RT-PCR results of the gene expression relative to GAPDH of terminal neural differentiation markers, βIII-tubulin and Map2, and brain development-related genes FoxG1 and SHH. This data may suggest that cells are ongoing to specialization processes from the commitment to neural progenitors. All nuclei were counterstained with DAPI. The scale bar is 50μm. **P<0.01.

We differentiated the iNSCs using adhesion-culture conditions with serum free-basal media. Differentiated cells formed a cluster in the middle with a web-like network with neuronal and glial morphologies. We detected the immunoreactivity of βIII-tubulin, MAP2, FOXG1, SHH, GFAP, and NeuN/Fox3 in the cells forming the web (Fig 3) for neurons and glia. Cells in the cluster were Sox2, Sox1, and CD90 positive, indicating that those cells retained stemness even after 21 days of differentiation. There was a significant increase in βIII-tubulin expressions in the cells derived from iNSCs produced with Method 2 vs. Method 1 (p = 0.0024); Method 4 vs. Method 3 (p = 0.0059); Method 3 vs. Method 2 (p = 0.0018) and Method 4 vs. Method 1 (p = 0.0079).

**Fig 4:**
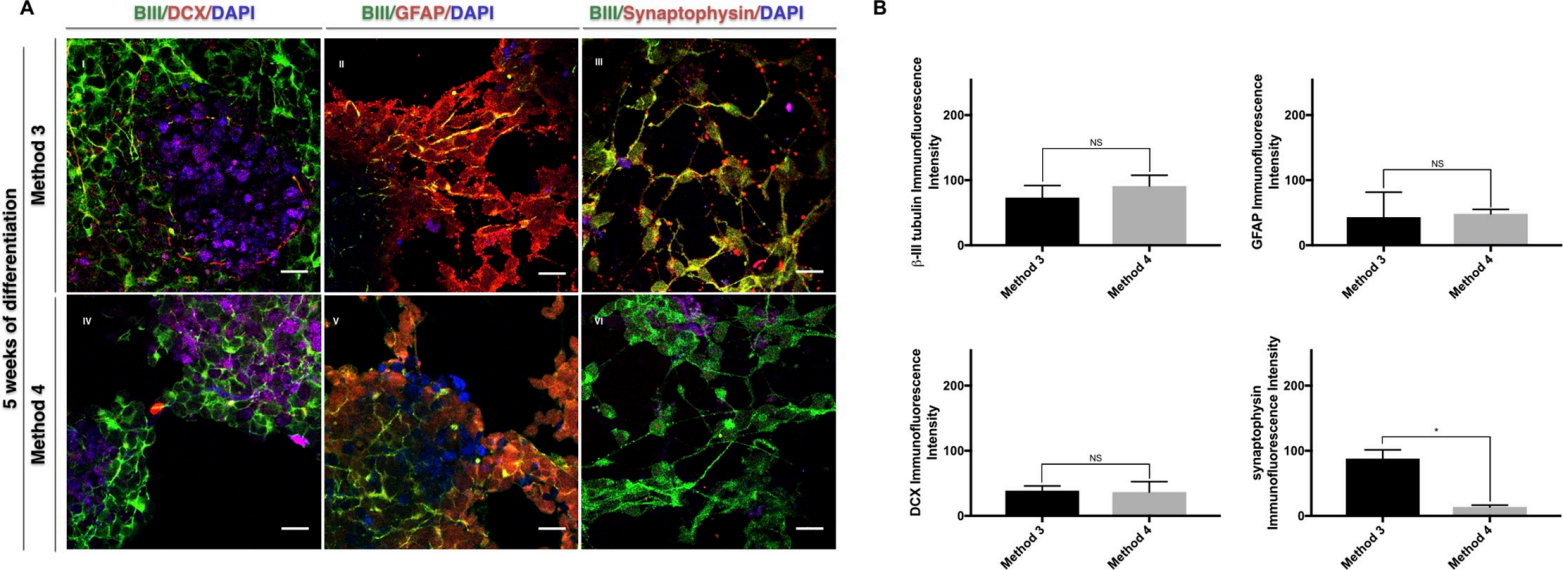
Effect of exosomes from pluripotent cells on the differentiation of transdifferentiation of MSCs to INSCs. Panel A shows the immunostaining of the NSCs-MSCs differentiated within four weeks, which express neurons and glial markers DCX, GFAP, βIII-tubulin, and synaptophysin method 3 and method 4. Panel B shows the difference between the immunofluorescence intensities of the immunostaining in panel A. All nuclei were counterstained with DAPI. The scale bar is 25μm. *P<0.05.

We also found immunoreactivity for doublecortin (DCX) and synaptophysin (Fig 4A and 4B). The synaptophysin protein expression was significantly different between iNSCs produced with methods 3 and 4 (p = 0.0149) and mainly detected in the areas where the cells showed web-like networks.

## Discussion

This study is to report a potential to produce NSCs like cells from adult human MSCs without animal products such as serum and gene transfection. This technology may provide us autologous cell materials for cell therapies of neurological disorders and in vitro disease models for drug screening and personalized medicine with a safe, time-saving, and cost-effective manner [27–30].

Researchers reported iNSCs could survived, migrated, differentiated, and improved behaviors after engraftment in Parkinson's disease models [29]. In closed head injury murine models, iNSCs and neural cells derived iNSCs modulated complement activation to protect neurons [28], and released neurotrophic factors, and modulated the host's immune response [17]. It is shown that iNSCs had a capability to integrate the host brain [16]. Upon transplantation, iNSCs differentiated into neural cells and improved the behavior of mouse stroke models without the development of tumors [18]. The researchers showed that iNSCs were safer than iPS cells because of the lack of tumorigenesis [18, 30], and even iNSCs repressed tumor regrowth [27].

Over a decade ago, our group reported that BrdU treatment facilitated trans-differentiation of bone marrow-derived MSCs into neural cells when they were co-cultured with differentiated NSCs or transplanted into the brain of animals [1]. We also demonstrated that the overexpression of the ES cell gene, NANOG, increased expression of OCT4 and SOX2 and potency of MSCs [2, 3]. Later, the Japanese group produced iPS cells from skin blasts by over expressions of Oct4, Sox2, Klf4, and c-Myc [4]. However, the use of c-Myc and viral transfection prevent the use of iPS cells in the clinic. Another approach used a plasmid with the Epstein-Barr virus (EBV) promotor to induce transcription factors Oct4, Sox2, Klf4, c-Myc, and Lin28 [7], which did not require the use of a virus, but the use of tumorigenic genes still pose problems. Evidence suggests that the different origins of the material cells, as well as the various combinations of transcription factors, affect the efficiency of the reprogramming and cell conversion [31]. For example, iPS cells can be produced from neural progenitors without using Sox2 [5]. However, there is no point in generating iPS cells from cells isolated from neural tissue because they are not easily accessible. Although many researchers have attempted to transdifferentiate somatic cells directly to neural cells avoiding issues associated with iPS cells, improvements are still needed.

Transdifferentiation of iNSCs may require less time than the production of NSCs through the production of iPS cells. The iPS cells require the downregulation of ES cell genes, such as Nanog and Oct4, for their differentiation [32]. Recently, a cost-effective method to produce iNSCs directly from fibroblasts was reported, though it may have safety issues because of the use of transgenes [11]. This issue is also evident in the transdifferentiation of human umbilical cord blood cells to iNSCs with over expressions of Sox2 and HMGA2 [12]. An alternative method to produce iNSCs was to use small molecules [14], which may be safer, but the resulting cells required feeder cells for expansion, indicating not fully functional NSCs. Another similar approach was a modulation of the TGF-*β* signaling pathway, which facilitated the development of cells with neuronal-like morphology directly from MSCs but lacked glial cell morphologies [15]. The contaminations of any Xeno- and transgene-materials, which might occur in any of the approaches mentioned above, compromise future therapeutic uses of the resulting cells. Thus, we need to develop Xeno- and transgene-free technology to produce NSCs from somatic cells.

In the current study, we produced iNSCs, which express NSC markers, from MSCs by the treatment with exosomes derived from NSCs and inhibition of TGF-*β* signaling. We found a mixed population of iNSCs and MSCs, which adhered to the surface of the plate with a fibroblastic-like shape and expressed the MSC biomarker CD73 in Method 1. The decitabine treatment (Method2) increased spheroid formation. The reduction of epigenetic restriction by decitabine might improve the trans-differentiation of MSCs. On the other hand, the addition of exosome treatment from iPS cells did not enhance the trans-differentiation of MSCs to iNSCs in any way. Treatment with iPS-derived exosomes may have some effects on the differentiation, but it would be better to avoid using them since they may contain a tumor gene used to create iPS cells and ES cell factors to cause many problems.

The iNSCs resulting from all the methods were capable of self-renewal and possessed multipotency to differentiate into different types of cells expressing neural cell markers. The results suggest that the combination treatments of NSC derived exosomes, TGF-*β* inhibitor, and decitabine (Method 2), may be the most efficient way to produce iNSCs from MSCs. In our cocktail, we used the TGF-*β* inhibitor SB431552 in all the Methods. Inhibition of TGF-*β* and BMP signaling pathways is also used to transdifferentiation of MSCs [33]. TGF-*β* signaling is essential for commitment to the mesenchymal cell lineage, and it is vital for their terminal differentiation because the downstream effectors are members of the SMAD family. Thus, the inhibition of the TGF-*β* may have impaired the commitment of MSCs and improved the reprogramming of the cells.

Thus, the mechanism of transdifferentiation observed in the current study could be explained as follows.

First, we used adult MSCs, which possess multipotency, self-renewal capabilities with less epigenetic restriction. Second, inhibition of SMAD signaling by the TGF-*β* inhibitor SB431552 suppressed mesenchymal cell lineage in MSCs. Third, we used decitabine, which is an FDA approved epigenetic modification drug for cancer therapy. It may have removed the cell commitment. Fourth, the introduction of neural cell linage commitment by using NSC derived exosomes, which contain neural factors.

## Conclusion

Here we show that MSCs can be reprogrammed to become iNSCs and bypass the traditional intermediate stage involving the production of iPS cells. This study demonstrates that the combination treatment of NSCs derived exosomes and TGF-*β* inhibitor allowed MSCs to transdifferentiate into iNSCs. A decitabine treatment, which removed MSC's epigenetic commitment, enhanced the efficiency of generating iNSCs. This Xeno and transgene-free transdifferentiation method may overcome the issues associated with iPS cells, such as tumorigenesis. It is also a time-efficient way to develop neural cells since it skips an inefficient procedure to produce iPS cells and a tedious differentiation process. Our iNSCs transdifferentiated from MSCs can expand in suspension cultures for multiple passages and differentiate into cells expressing neural markers upon the differentiation. We hope this technology opens a door for the development of neuroregenerative therapies and the production of *in vitro* neurological disease models for personalized medicine and drug screening.

## Supporting information

S1 Fig Comparison of cell markers between MSCs and iNSCs. Panel A shows the original MSCs positive staining for CD105, CD90, and CD73 and negative for CD45. Panel B shows the FACS for the MSCs and the iNSCs produced using the four methods for CD105, CD90, and CD73 and CD45. MSCs show high expression levels of their three biomarkers; however, the four methods used to produce iNSCs only express CD90. Panel C shows that differentiated MSCs were immunopositive for FABP4, Aggrecan, and osteocalcin, which are the markers for adipocytes, chondrocytes and osteocytes, respectively. The F-actin is stained with phalloidin to show the cell structure of those cells. Nuclei are counterstained with DAPI.
(TIFF)

S2 Fig Characterization of iNSCs-MSCs. The resultant cells from the four methods expressed NSC markers after expanded on adherent conditions on laminin. Panel A shows the immunoreactivity for neural stem cell markers MSI1, Sox1, Nestin, Pax6, GFAP, as well as for stemness markers, Oct4 and Nanog after 4 passages in the culture. Panel B shows the differences in the fluorescence intensity neural cell markers, MSI1, Sox1 and Nestin, and Nanog. Data suggest that there may be significant differences in the expression of neural cell marker, sox1 and nestin and stem cell markers, Oct4 and Nanog, between iNSC-MSCs produced from methods 3 and 4. All nuclei were counterstained with DAPI. The scale bar is 50 μm.
(TIFF)

S3 Fig Exosomes were used as a cell-commitment source for the production of cells expressing neural markers. Confocal micrograph (S3A: I&III), and flow cytometry (S3A: II&IV) of NSCs derived exosomes, and iPS cells derived exosomes, stained with a dye specific for proteins of extracellular vesicles/exosomes ExoGlow-protein green (ExoGreen). The arrows at the confocal images point to clumps of exosomes. Panel B shows the internalization of the NSCs exosomes labeled with the ExoGreen dye into the cultures of MSCs. The arrow points to the clumps of exosomes. The scale bar of the confocal image is 10μm.
(TIFF)
