# Supplementary figures and images for: Xeno- and transgene-free reprogramming of mesenchymal stem cells toward the cells expressing neural markers using exosome treatments
Source: PLoS One. 2020 Oct 13;15(10):e0240469. doi: 10.1371/journal.pone.0240469 (PMC7553345; doi:10.1371/journal.pone.0240469)

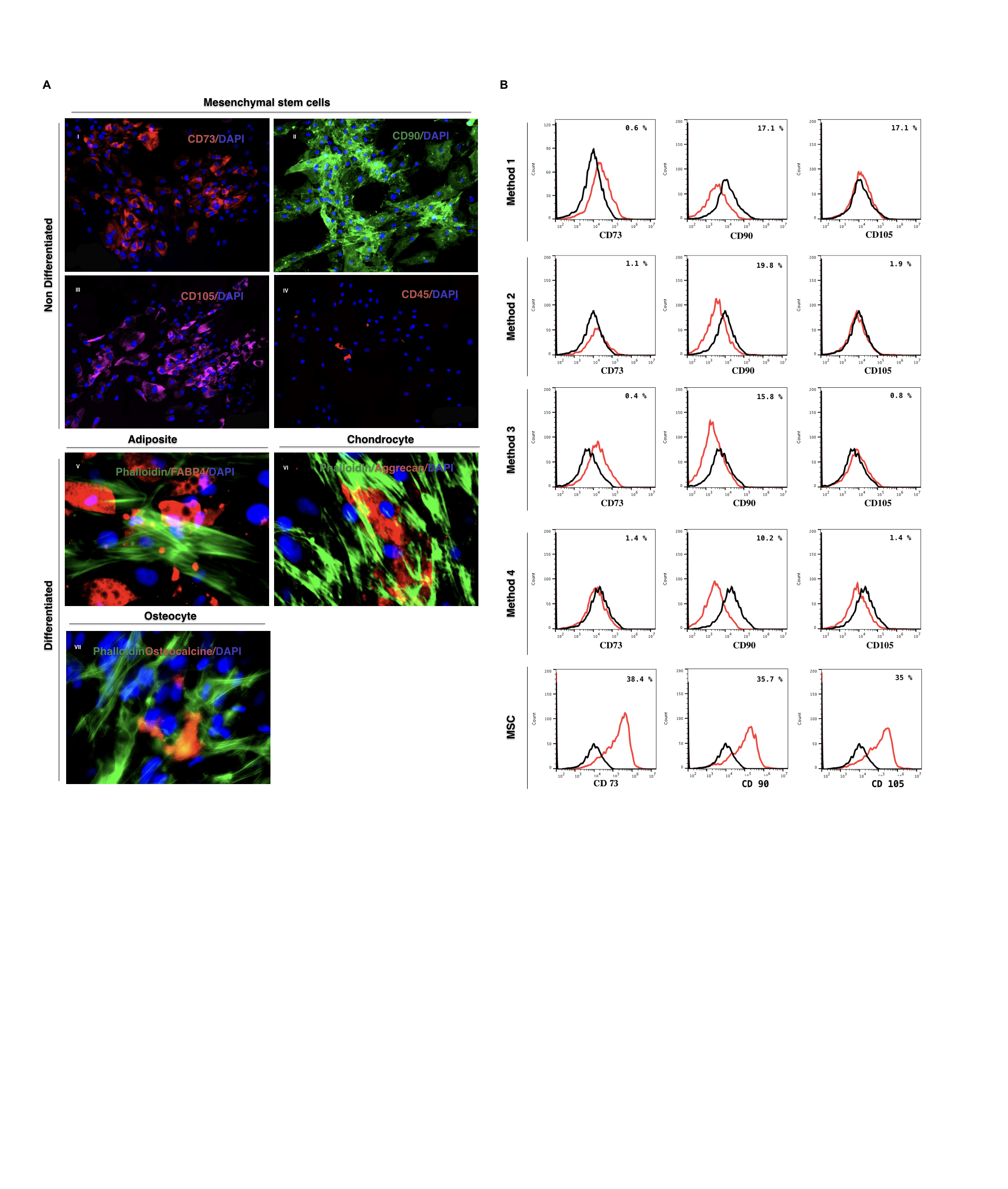

Supplement: S1 Fig — Panel A shows the original MSCs positive staining for CD105, CD90, and CD73 and negative for CD45. Panel B shows the FACS for the MSCs and the iNSCs produced using the four methods for CD105, CD90, and CD73 and CD45. MSCs show high expression levels of their three biomarkers; however, the four methods used to produce iNSCs only express CD90. Panel C shows that differentiated MSCs were immunopositive for FABP4, Aggrecan, and osteocalcin, which are the markers for adipocytes, chondrocytes and osteocytes, respectively. The F-actin is stained with phalloidin to show the cell structure of those cells. Nuclei are counterstained with DAPI. (TIFF) [file pone.0240469.s001.tiff]

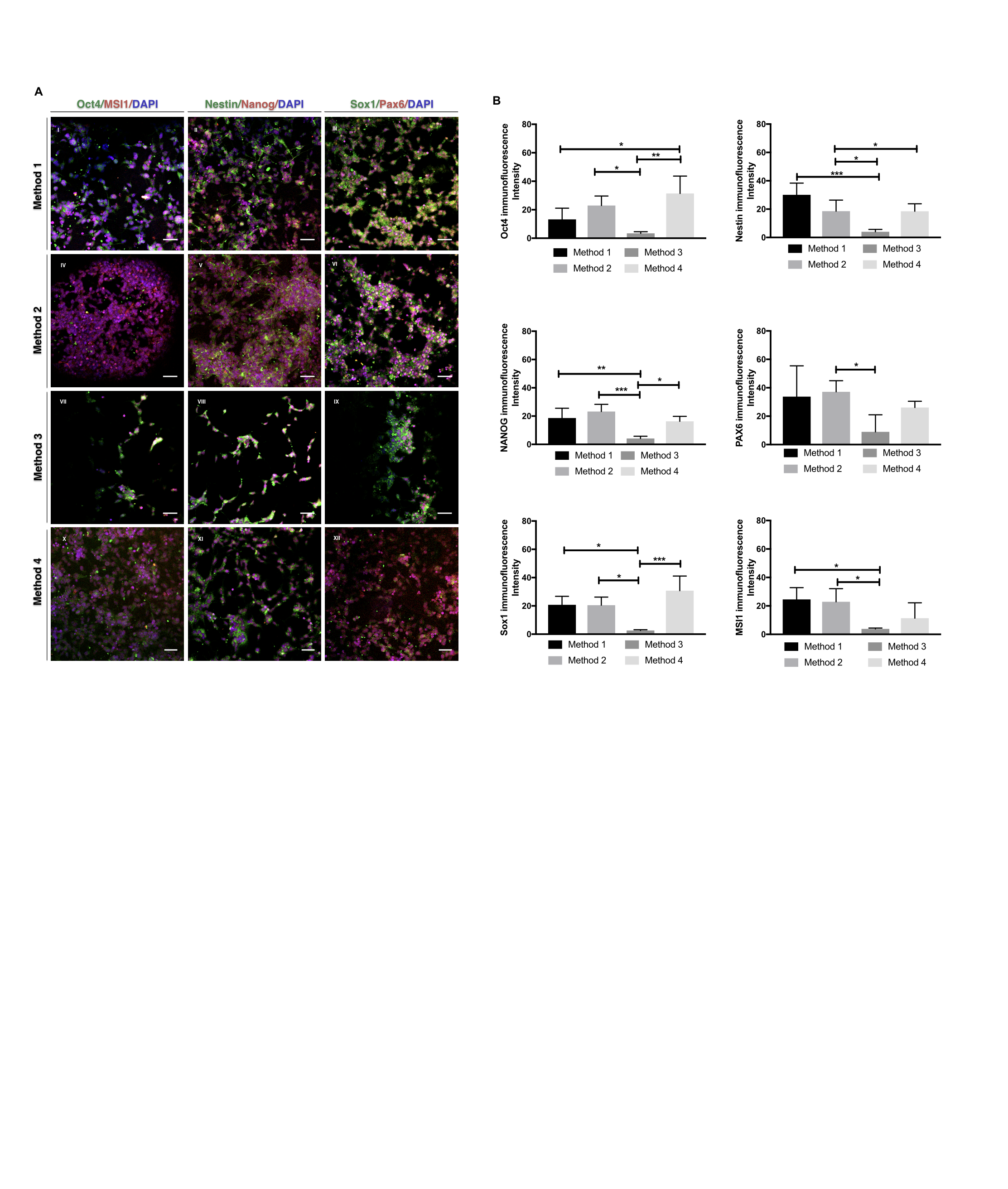

Supplement: S2 Fig — The resultant cells from the four methods expressed NSC markers after expanded on adherent conditions on laminin. Panel A shows the immunoreactivity for neural stem cell markers MSI1, Sox1, Nestin, Pax6, GFAP, as well as for stemness markers, Oct4 and Nanog after 4 passages in the culture. Panel B shows the differences in the fluorescence intensity neural cell markers, MSI1, Sox1 and Nestin, and Nanog. Data suggest that there may be significant differences in the expression of neural cell marker, sox1 and nestin and stem cell markers, Oct4 and Nanog, between iNSC-MSCs produced from methods 3 and 4. All nuclei were counterstained with DAPI. The scale bar is 50 μm. (TIFF) [file pone.0240469.s002.tiff]

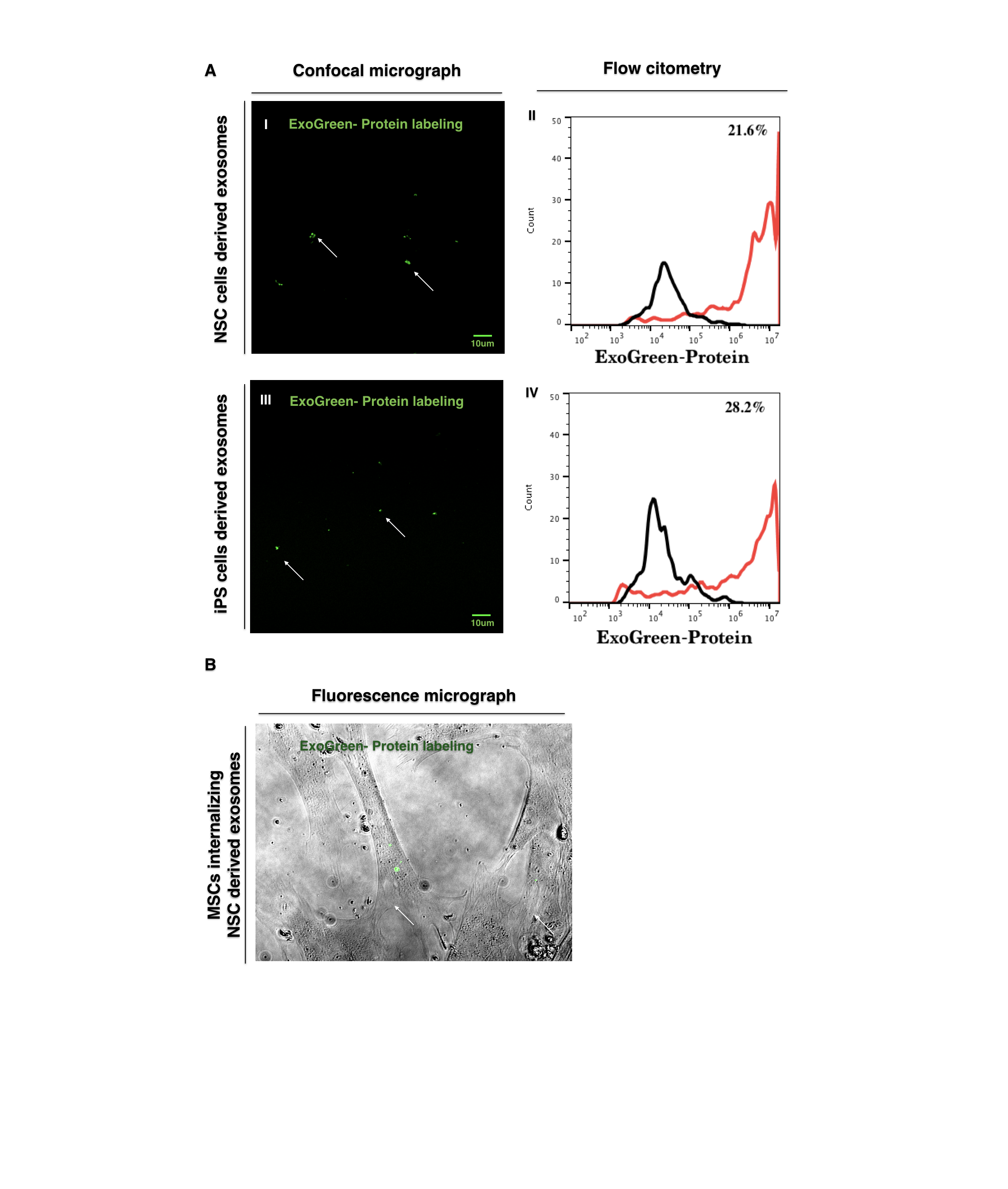

Supplement: S3 Fig — Confocal micrograph (S3A: I&III), and flow cytometry (S3A: II&IV) of NSCs derived exosomes, and iPS cells derived exosomes, stained with a dye specific for proteins of extracellular vesicles/exosomes ExoGlow-protein green (ExoGreen). The arrows at the confocal images point to clumps of exosomes. Panel B shows the internalization of the NSCs exosomes labeled with the ExoGreen dye into the cultures of MSCs. The arrow points to the clumps of exosomes. The scale bar of the confocal image is 10μm. (TIFF) [file pone.0240469.s003.tiff]
